# Molecular characterization and zoonotic potential of *Cryptosporidium* spp. and *Giardia duodenalis* in humans and domestic animals in Heilongjiang Province, China

**DOI:** 10.1186/s13071-024-06219-3

**Published:** 2024-03-25

**Authors:** Yaru Hao, Aiqin Liu, He Li, Yiyang Zhao, Lan Yao, Bo Yang, Weizhe Zhang, Fengkun Yang

**Affiliations:** Department of Parasitology, Harbin Medical University, Harbin, 150081 Heilongjiang China

**Keywords:** *Cryptosporidium* spp., *Giardia duodenalis*, Epidemiology, Domestic animals, Humans

## Abstract

**Background:**

Cryptosporidiosis and giardiasis are significant parasitic diseases shared between humans and domestic animals. Due to the close contact between humans and domestic animals in rural areas, it is important to consider the potential transmission of zoonotic parasites from infected domestic animals to humans. This investigation aimed to determine the prevalence and molecular characteristics of *Cryptosporidium* spp. and *Giardia duodenalis* in domestic animals and villagers.

**Methods:**

A total of 116 fecal samples from villagers and 686 fecal samples from domestic animals in Heilongjiang Province, China, were analyzed for two parasites using nested polymerase chain reaction (PCR) targeting various genetic loci and DNA sequence analysis of the PCR products.

**Results:**

By sequence analysis of the *SSU* rRNA gene, the prevalence of *Cryptosporidium* in humans was 0.9% (1/116), with one species of *C. parvum* (*n* = 1) detected; among domestic animals, the prevalence was 2.6% (18/686), with five species identified: *C. suis* (*n* = 7) and *C. scrofarum* (*n* = 7) in pigs, *C. meleagridis* (*n* = 1) in chickens, *C. andersoni* (*n* = 1) in cattle, and *C. canis* (*n* = 2) in foxes. *C. parvum and C. canis* were further subtyped as IIdA19G1 and XXa4 on the basis of *gp60* gene. Regarding *G. duodenalis*, based on the *SSU* rRNA, *bg*, *gdh*, and *tpi* genes, the prevalence in domestic animals was 5.1% (31/608), with three assemblages identified: A (*n* = 1) in pigs, D (*n* = 1) in foxes, and E (*n* = 27) in geese, cattle, pigs, ducks, and sheep, along with mixed infection of A + E (*n* = 1) in one pig and B + E (*n* = 1) in one sheep. No *G. duodenalis* was detected in humans (0/116).

**Conclusions:**

The present results show that no overlap of subtypes between animals and villagers was found in *Cryptosporidium* spp. and *G. duodenalis*, indicating a minor role of domestic animals in infecting humans in this population. However, the presence of zoonotic protozoa in domestic animals highlights the need for special attention to high-risk individuals during close contact with domestic animals.

**Graphical Abstract:**

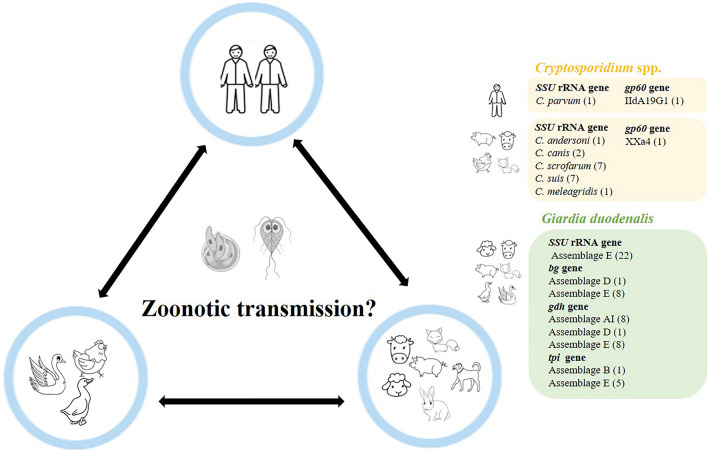

**Supplementary Information:**

The online version contains supplementary material available at 10.1186/s13071-024-06219-3.

## Background

*Cryptosporidium* spp. and *Giardia duodenalis* (*G. duodenalis*, also known as *G. lamblia* or *G. intestinalis*) are important protozoan parasites with primary clinical symptoms of diarrhea. These parasites are widespread and can infect humans, domestic animals, and wild animals. The cysts/oocysts of both parasites are shed in the feces of infected hosts and can be transmitted to new hosts via contaminated food, water, or direct contact with infected humans or animals [1, 2]. Both protozoa have a monoxenous life cycle, a low infective dose, and a short prepatent period, which increase their potential for transmission in human and animal hosts [3]. Some families keep domestic animals in their yards and share public places with them, increasing the potential for zoonotic transmission [2].

The oocysts of many *Cryptosporidium* species are difficult to distinguish from one another, as they share similar morphological characteristics. Therefore, molecular methods are essential for identifying *Cryptosporidium* species, genotypes, and subtypes that can identify organisms and trace the infection source and transmission routes [4]. Targeting the small subunit of ribosomal RNA (*SSU* rRNA) is a useful tool for *Cryptosporidium* species identification [5]. To date, extensive genetic variation has been identified within the *Cryptosporidium*, with at least 49 valid *Cryptosporidium* species and more than 120 genotypes being recognized [1, 6]. Among them, 23 species and two genotypes have been identified in humans, and *C. hominis* and *C. parvum* are the most common species, accounting for 95% of human cases [1, 7, 8]. In addition, with the development of whole-genome sequencing, a gene encoding 60-kDa glycoprotein (*gp60*) has been employed as a marker to subtype 23 *Cryptosporidium* species and two genotypes [9]. Similarly, genotyping of *G. duodenalis* is crucial for epidemiological studies, which are mainly performed using sequence analysis of polymerase chain reaction (PCR) products from genes, such as *SSU* rRNA gene, β-giardin (*bg*), glutamate dehydrogenase (*gdh*), and triosephosphate isomerase (*tpi*) [10]. Using multiple markers ensures more reliable genotyping results and helps detect mixed infections with different assemblages in humans or animals [11]. *G. duodenalis* is classified into eight assemblages (A–H) based on protein or DNA polymorphisms, and assemblages A and B are considered zoonotic, which primarily cause infections in humans and mammals. The other six assemblages (C–H) are host specific, however, assemblages C–F are also occasionally identified in humans [1]. Only assemblage A can be further divided into three sub-assemblages (AI, AII, and AIII), while no recognized nomenclature exists for assemblages B–H [10].

The potential transmission of these two parasites among humans and animals living in close proximity has been sparsely reported. In Côte d’Ivoire, the occurrence of *Cryptosporidium* and *G. duodenalis* in humans and free-ranging animals living in remote rural zones provided evidence about the potential role of these domestic animals living closely with humans in the environmental dissemination and transmission of these anthropozoonotic parasites to humans [12]. Conversely, in Western Uganda, no evidence of potential transmission of *Cryptosporidium* and *G. duodenalis* was found among closely cohabiting people, domestic animals, and wild nonhuman primates [13]. Meanwhile, in Álava, northern Spain, no molecular epidemiological evidence supported household transmission of zoonotic *Cryptosporidium* and *G. duodenalis* from pet dogs and cats [14]. On the basis of the studies in different regions, it can be concluded that the occurrence and potential transmission of *Cryptosporidium* and *G. duodenalis* can vary depending on the specific geographical location and the interactions between humans and animals.

In China, there are also few data available for molecular epidemiological studies on both parasites in humans and domestic animals coexisting in the same habitat. In Heilongjiang Province, domestic animals such as cattle, goats, sheep, pigs, chickens, and other species are raised in an agricultural setting to produce various commodities—usually food (meat, organs, eggs, dairy products), as well as hair or wool [15]. Due to the close contact between humans and domestic animals, there are concerns about the potential transmission of the zoonotic species/assemblages of these two parasites from infected domestic animals to humans. The aim of the study was to determine the prevalence and distribution of species, genotypes, and subtypes of *Cryptosporidium* and *G. duodenali* assemblages and sub-assemblages in humans and domestic animals and understand the role of animals in the environmental dissemination of zoonotic *Cryptosporidium* and *G. duodenali*s.

## Methods

### Sources and collections of fecal samples

**Fig. 1:**
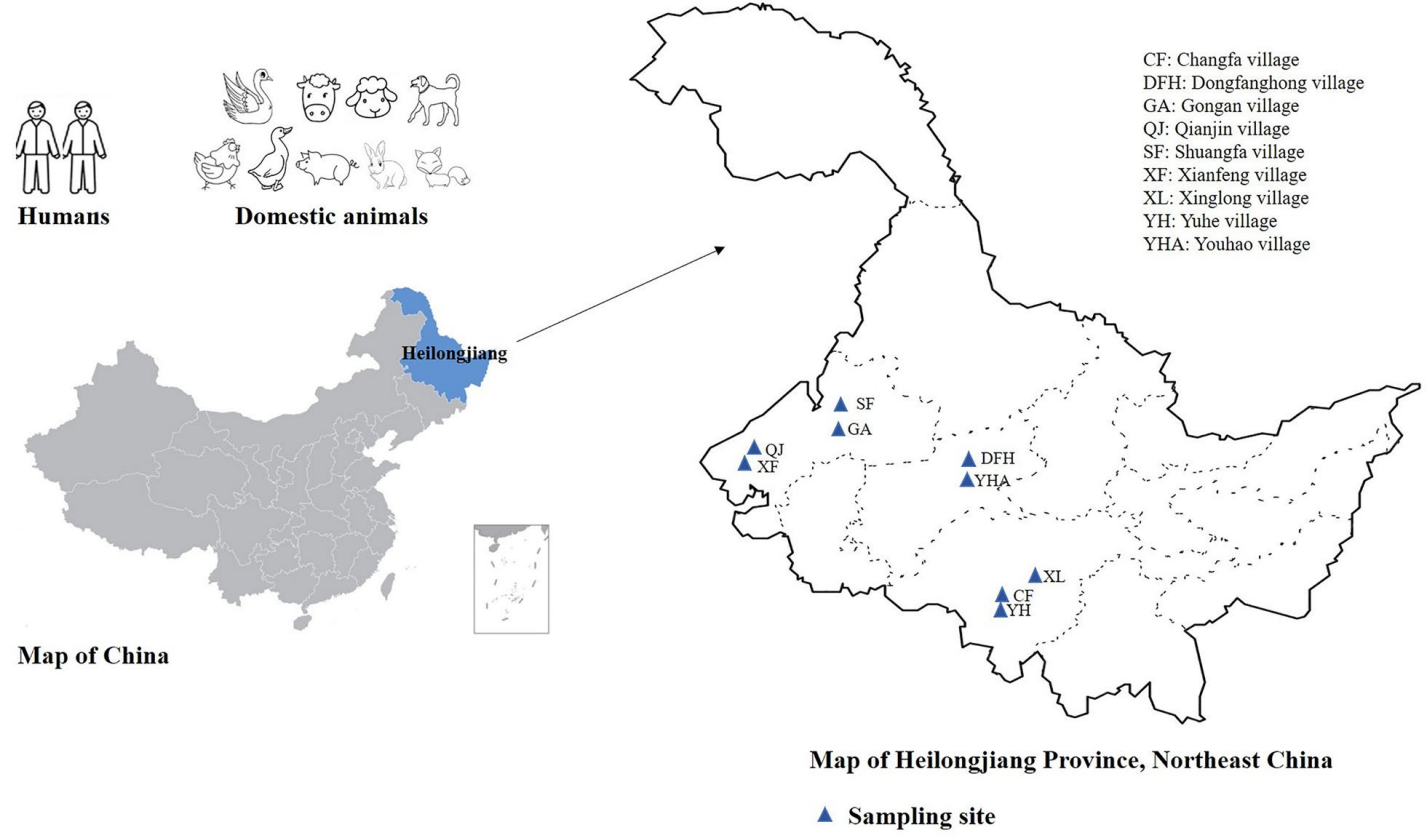
Sampling sites for enteric protist samples from villagers and domestic animals in Heilongjiang Province, China

From November 2020 to December 2022, a total of 802 fecal samples (approximately 5–10 g) were collected from humans and animals (one sample from each) from nine villages in Heilongjiang Province, China (Fig. 1, Table 1). For families with domestic mammals and poultry, the participants were randomly selected to provide individual fecal samples including animals. A total of 68 households were included. Among these households, 5 only provided fecal samples of animal origin, and 20 households only provided samples of human origin. None of the humans and domestic animals had any apparent clinical symptoms of diarrhea at the time of sampling. Each fecal sample was collected from the upper portion of fresh feces deposited on the ground after defecation by using disposable gloves and placed into 50 ml sterile containers individually. All the samples were transported to the laboratory in a cooler with ice packs within 24 h and stored in refrigerators at 4 ℃ (≤ 2 days) or −20 ℃ (> 2 days) prior to using in the subsequent molecular analysis.

**Table 1:** Fecal sample collection details from humans and domestic animals in nine villages, Heilongjiang Province, China

Location	Geographical coordinate	Altitude (*m*)	No. of samples												Total (*n*)
			Human	Domestic mammal								Poultry			
			Villager	Cattle	Dog	Fox	Goat	Pig	Raccoon dog	Rabbit	Sheep	Chicken	Duck	Goose	
CF	126.42′E, 45.96′N	146	3	0	3	0	0	50	0	0	0	14	0	10	80
DFH	127°05′E, 47°31′N	232	26	8	7	0	12	28	0	0	0	18	1	0	100
GA	125°04′E,48°05′N	255	17	0	1	0	0	0	0	0	30	10	1	0	59
QJ	124°57′′E,48°24′N	196	15	24	0	0	0	0	0	0	0	20	0	0	59
SF	125°09′E,48°30′N	212	21	9	2	0	2	22	0	2	0	80	8	13	159
XF	125°33′E, 48°33N	259	12	0	0	0	0	0	0	0	0	10	0	0	22
XL	126.90′E, 45.29′N	186	3	0	1	0	0	39	0	0	0	3	0	2	48
YH	128°22′E,45°23′N	198	10	3	2	0	0	11	0	0	0	42	0	9	77
YHA	126°48′E,47°08′N	207	9	7	3	46	0	10	3	0	15	56	0	49	198
Total	–	–	116	51	19	46	14	160	3	2	45	253	10	83	802

*CF* Changfa village, *DFH* Dongfanghong village, *GA* Gongan village, *QJ* Qianjin village, *SF* Shuangfa, *XF* Xianfeng, *XL* Xinglong, *YH* Yuhe village, *YHA* Youhao village

### Processing of fecal samples and DNA extraction

The fecal samples of herbivores needed to be processed by sieving the crude fiber and impurities in the samples and concentrating for 10 min at 1500 g. Meanwhile, the fecal samples of the other animals and humans were simply washed twice with distilled water by centrifugation to concentrate the samples. Then, genomic DNA (gDNA) was extracted from approximately 200 mg of processed fecal samples using the QIAamp DNA Mini Stool Kit (Qiagen, Hilden, Germany), following the manufacturer’s recommended procedure. The extracted DNA was then stored at −20 °C until it was used for PCR amplification.

### PCR amplification

*Cryptosporidium* was detected by nested PCR amplification of the *SSU* rRNA gene fragment of ∼ 830 base pairs (bp) [16]. Further, *C. parvum*-, *C. meleagridis*-, and *C. canis*-positive samples were subtyped by nested PCR amplification of the *gp60* gene. Different subtyping tools based on *gp60* gene were utilized for subtyping three *Cryptosporidium* species [17–19].

The assemblages and sub-assemblages of *G. duodenalis* were identified by a nested PCR to amplify the *SSU* rRNA (290 bp) [20], *bg* (511 bp) [21], *gdh* (530 bp) [22], and *tpi* (530 bp) genes [23]. In addition, to detect mixed infections of different *G. duodenalis* assemblages within the same sample, specific nested PCRs were performed to amplify the *tpi* gene with fragment sizes of approximately 330 bp (assemblage A), 400 bp (assemblage B), and 390 bp (assemblage E) [24, 25].

TaKaRa Taq DNA polymerase (TaKaRa Bio Inc., Tokyo, Japan) was utilized for all PCR amplification. Each PCR analysis included positive and negative controls, and each sample was subjected to at least two PCR analyses at each genetic site. All secondary PCR products were separated by 1.5% agarose gel electrophoresis stained with GelStain (TransGen Biotech., Beijing, China).

### DNA sequencing and nucleotide sequence analysis

All positive secondary PCR products of the expected size were subjected to sequencing using the secondary PCR primers on an ABI PRISMTM 3730 DNA analyzer (Applied Biosystems, Waltham, MA, USA) with the BigDye Terminator v3.1Cycle Sequencing Kit (Applied Biosystems). The accuracy of the nucleotide sequence was confirmed by two-directional sequencing. If novel nucleotide sequences were obtained from certain DNA samples, two additional new PCR products were sequenced as necessary. Nucleotide sequences obtained in the present study were aligned and analyzed with each other and reference sequences that were downloaded from GenBank using the Basic Local Alignment Search Tool (BLAST) (http://www.ncbi. nlm.nih.gov/blast/). Generated DNA consensus sequences were aligned to appropriate reference sequences using the MEGA 5 software (http://www.megasoftware.net) to identify the species/subtypes of *Cryptosporidium* and the assemblages/sub-assemblages of *G. duodenalis*.

The representative nucleotide sequences obtained in the present study were deposited in the GenBank database under the following accession numbers: human-derived *Cryptosporidium* isolates—OR357663 (*SSU* rRNA) and OR353407 (*gp60*); animal-derived *Cryptosporidium* isolates—OR357660 to OR357662, OR357664 to OR357666 (*SSU* rRNA), and OR353406 (*gp60*); animal-derived *G. duodenalis* isolates—OR359371 to OR359372 (*SSU* rRNA), OR353408 to OR353412 (*bg*), OR360610 to OR360618 (*gdh*), and OR353413 to OR353416 (*tpi*).

### Phylogenetic analysis

The phylogenetic tree was constructed on the basis of the neighbor-joining (NJ) method and the Kimura-2-parameter model using the program MEGA 5. To assess the tree’s reliability, a bootstrap analysis with 1000 replicates was performed. Reference sequences from GenBank were downloaded, and the sequences were labeled with National Center for Biotechnology Information (NCBI) accession number, the host origin, and the country.

## Results

### Prevalence of *Cryptosporidium* spp. and *G. duodenalis*

Using PCR amplification and sequence analysis, *Cryptosporidium* was found in humans and domestic animals, while *G. duodenalis* was only found in some domestic animals. The prevalence of *Cryptosporidium* was 0.9% (1/116) in humans, whereas the overall prevalence was 2.6% (18/686) in domestic animals, with 5.0% (17/340) in domestic mammals, including cattle (2.0%, 1/51), foxes (4.3%, 2/46), and pigs (8.8%, 14/160), with 0.3% (1/346) in poultry, including chickens (0.4%, 1/253). Meanwhile, *Cryptosporidium* was absent in dogs, goats, raccoon dogs, rabbits, sheep, ducks, and geese (Table 2). For *G. duodenalis*, in domestic animals the overall prevalence was 4.5% (31/686). The prevalence was 8.2% (28/340) in domestic mammals, including cattle (9.8%, 5/51), foxes (2.2%, 1/46), pigs (3.1%, 5/160), and sheep (28.8%, 17/59), and the prevalence was 0.9% (3/346) in poultry, including ducks (10.0%, 1/10) and geese (2.4%, 2/83) (Table 2). Meanwhile, *G. duodenalis* was absent in humans and domestic animals (dogs, goats, raccoon dogs, rabbits, and chickens).

**Table 2:** Prevalence and distribution of *Cryptosporidium* species/subtypes and *G. duodenalis* assemblages/sub-assemblages in humans and domestic animals

Host (*n*)		*Cryptosporidium*			*G. duodenalis*				
		Positive no. (%)	*SSU* rRNA (*n*)	*gp60* (*n*)	Positive no. (%)	*SSU* rRNA (*n*)	*bg* (*n*)	*gdh* (*n*)	*tpi*^a^ (*n*)
Humans	Villager (116)	1 (0.9)	*C. parvum* (1)	IIdA19G1 (1)	0				
Domestic animals									
Domestic mammals	Cattle (51)	1 (2.0)	*C. andersoni* (1)		5 (9.8)	E (4)	E (5)	E (4)	E (2)
	Dog (19)	0			0				
	Fox (46)	2 (4.3)	*C. canis* (2)	XXa4 (2)	1 (2.2)		D (1)	D (1)	
	Goat (14)	0			0				
	Pig(160)	14 (8.8)	*C. scrofarum* (7); *C. suis* (7)		5 (3.1)	E (1)	E (1)	AI (2); E (2)	E (1)
	Raccoon dog (3)	0			0				
	Rabbit (2)	0			0				
	Sheep (45)	0			17 (28.8)	E (17)		E (2)	B (1); E (2)
	Subtotal	17 (5.0)	*C. andersoni* (1); *C. cains* (2); *C. scrofarum* (7); *C. suis* (7)		28 (8.2)	E (22)	E (6); D (1)	AI (2); D (1); E (8)	B (1); E (5)
Poultry	Chicken (253)	1 (0.4)	*C. meleagridis* (1)		0				
	Duck (10)	0			1 (10)			E (1)	
	Goose (83)	0			2 (2.4)		E (2)		
	Subtotal	1 (0.3)	*C. meleagridis* (1)		3 (0.9)		E (2)	E (1)	
Total	Human (116)	1 (0.9)	*C. parvum *(1)	IIdA19G1 (1)	0				
	Domestic animal (686)	18 (2.6)	*C. andersoni* (1); *C. cains* (2); *C. scrofarum* (7); *C. suis* (7); *C. meleagridis* (1)	XXa4 (2)	31 (4.5)	E (22)	E (8); D (1)	AI (2); D (1); E (9)	B (1); E (5)

^a^At the *tpi* locus, all *G. duodenalis*-positive samples were only sequenced successfully using assemblage-specific primers

### Molecular characteristics of *Cryptosporidium* spp. isolates

**Fig. 2:**
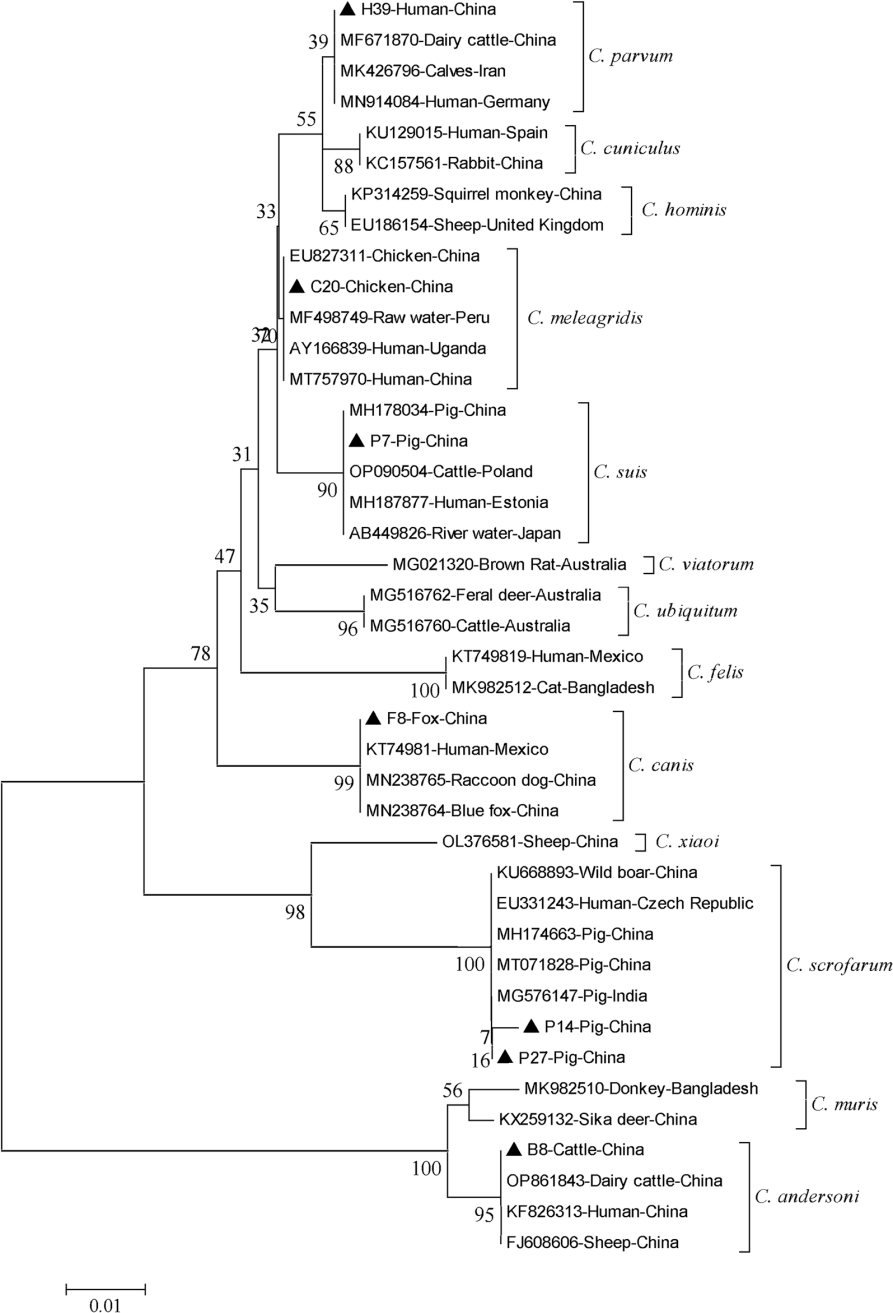
The phylogram of *Cryptosporidium* spp. was inferred on the basis of the *SSU* rRNA gene nucleotide sequences. The evolutionary relationship of *Cryptosporidium* spp. was constructed by the NJ method and Kimura 2-parameter model. The numbers on the branches are percent bootstrapping values from 1000 replicates. Each reference sequence is identified by its accession number, host, and country. The triangles filled in black represent the representative sequence obtained in this study. Evolutionary analyses were conducted in MEGA 5

On the basis of sequence analysis of the *SSU* rRNA gene, a total of six species/genotypes of *Cryptosporidium* were identified out of 19 isolates, including *C. parvum* (*n* = 1) in humans, *C. suis* (*n* = 7) and *C. scrofarum* (*n* = 7) in pigs, *C. meleagridis* (*n* = 1) in chickens, *C. andersoni* (*n* = 1) in cattle, and *C. canis* (*n* = 2) in foxes (Table 2). The sequences of the *SSU* rRNA gene for the *C. parvum*, *C. suis*, *C. meleagridis*, *C. andersoni*, and *C. canis* in this study are 100% identical to reference sequence deposited in GenBank. Among the seven *C. scrofarum* isolates from pigs, six showed complete sequence identity with a sequence (MG576147) from a pig in India. One isolate showed 98.9% similarity to *C. scrofarum* (MH174663) obtained from a Tibetan pig in Henan, China, with a single base difference (C528T) (Additional file 1: Table S1). Phylogenetic analysis of *SSU* rRNA nucleotide sequences confirmed the observations made during the sequencing analysis (Fig. 2).

**Fig. 3:**
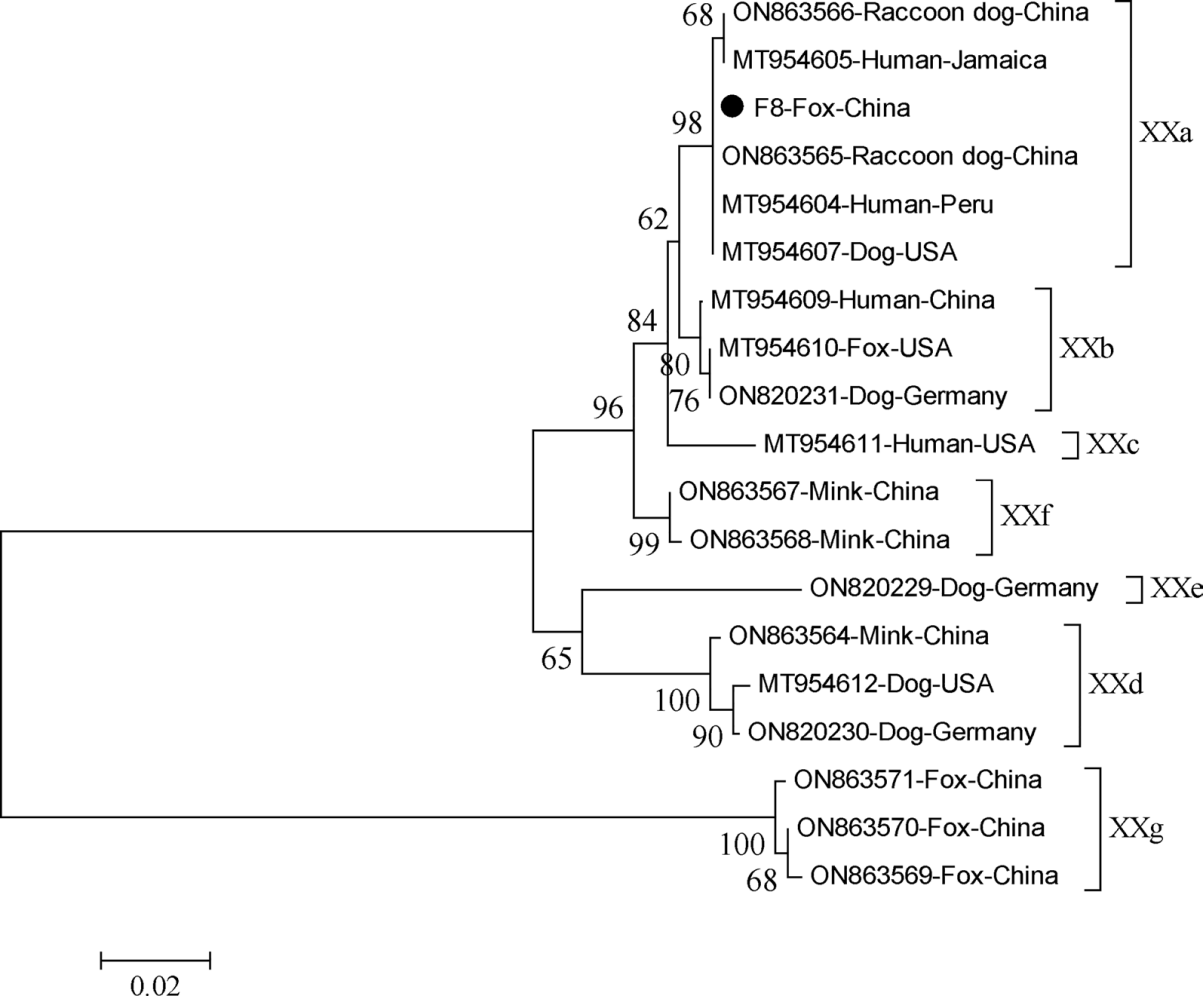
The phylogram of *Cryptosporidium* spp. was inferred on the basis of the nucleotide sequences of the *gp60* gene. The evolutionary relationship of *Cryptosporidium* spp. was constructed by the NJ method and Kimura 2-parameter model. The numbers on the branches are percent bootstrapping values from 1000 replicates. Each reference sequence is identified by its accession number, host, and country. The circle filled in black represents the representative sequence obtained in this study. Evolutionary analyses were conducted in MEGA 5

For subtyping of the *Cryptosporidium* species, DNA samples characterized as originating from *C. parvum*, *C. meleagridis*, and *C. canis* were further subjected to sequence analysis of the *gp60* gene. One *C. parvum* isolate was successfully sequenced and identified as subtype IIdA19G1. In addition, two *C. canis* isolates were successfully sequenced and both were identified as subtype XXa4. Phylogenetic analysis of the *gp60* nucleotide sequences revealed that *C. canis* subtype XXa4 sequences obtained from two foxes in this study, as well as sequences from human, raccoon dog, and dog downloaded from GenBank, were clustered together into one clade with high bootstrap value (Fig. 3).

### Molecular characteristics of *G. duodenalis* isolates

There are 31 *G. duodenalis*-positive samples based on four loci, and 22 *SSU* rRNA, 9 *bg*, 12 *gdh*, and 6 *tpi* gene sequences were obtained. Only one positive sample was successfully sequenced at all four loci, whereas the remaining samples were amplified at one to three loci (Additional file 2: Table S2). Sequence analysis results showed that assemblage A (*n* = 1) was identified in one pig, assemblage D (*n* = 1) in one fox, and assemblage E (*n* = 27) in pigs, cattle, sheep, ducks, and geese. Additionally, mixed infections were identified in one pig (A + E) and one sheep (B + E) (Additional file 2: Table S2).

The genetic diversity of *G. duodenalis* among assemblages E, D, and A was observed at the *bg*, *gdh*, and *tpi* loci. Within assemblage E, four representative sequences (E^#1^–E^#4^) having single nucleotide substitutions (SNPs) were obtained from one, two, three, and one isolates at the *bg* locus, respectively; six representative sequences (E^#5^–E^#10^) having SNPs were obtained from two, two, one, one, two, and one isolates at the *gdh* locus, respectively; and three representative sequences (E^#11^–E^#13^) with SNPs were obtained from one, two, and one isolates at the *tpi* locus, respectively. Within assemblage D, one representative sequence (D^#1^) was obtained from one isolate at the *bg* locus, and one representative sequence (D^#2^) was obtained from the same isolate at the *gdh* locus. Furthermore, within assemblage A, two representative sequences (AI^#1^ and AI^#2^) were obtained from two isolates at the *gdh* locus, both belonging to AI. Only one representative sequence (B^#1^) was obtained from one isolate at the *tpi* locus (Additional file 3: Table S3).

### Distribution of species/subtype of *Cryptosporidium* spp. and assemblage/sub-assemblage of *G. duodenalis* in humans and domestic animals in households

A total of 68 family households were investigated, and regardless of the host, *Cryptosporidium* and/or *G. duodenalis* were detected in 22.1% (15/68) of these households, as presented in Table 3. In 53.3% (8/15) of the households, *Cryptosporidium* was detected in human or domestic animal fecal samples. Additionally, in 73.3% (11/15) of the households, *G. duodenalis* was identified in domestic animals. Simultaneous infections by any of these two parasites in humans and domestic animals living together in the same environment were only demonstrated in one household: in the DFH-5 family, *Cryptosporidium* was detected in both one cow and its owner. However, this cow was found to be infected with *C. andersoni*, while the owner was infected with *C. parvum*. Simultaneous infections by the same species/subtypes of *Cryptosporidium* and/or assemblage of *G. duodenalis* in domestic animals in the same environment were demonstrated in nine households. For *Cryptosporidium*, in the DFH-3 family, *C. suis* was identified in *Cryptosporidium*-positive pigs (*n* = 4), which was also found in pigs (*n* = 3) from the CF-1 family. Additionally, in the CF-1 family, *C. scrofarum* was identified in pigs (*n* = 4). In the YHA-3 family, *C. canis* (subtype XXa4) was identified in *Cryptosporidium*-positive foxes (*n* = 2). For *G. duodenalis*, assemblage E was detected in *Giardia*-positive sheep (*n* = 14) in the GA-4 family and in geese (*n* = 2) in the YHA-2 family. Assemblage E was also detected in cattle (*n* = 2) from the YH-5 family and cattle (*n* = 2) from the DFH-2 family, pigs (*n* = 2) in the DFH-9 family, and sheep (*n* = 2) in the YHA-7 family. One pig from the DFH-9 family was infected with *Cryptosporidium* and *G. duodenalis* (Table 3).

**Table 3:** Distribution of *Cryptosporidium* species/subtypes and *G. duodenalis* assemblages/sub-assemblages in humans and domestic animals by household

Household	Human	Domestic animal	
	*Cryptosporidium* (ID)	*Cryptosporidium* (ID)	*G. duodenalis* (ID)
YH-3		*C. meleagridis* (C20)	
YH-5			E (B18, B19)
YH-7		*C. scrofarum* (P30)	
DFH-2			E (B2, B3)
DFH-3		*C. suis* (P7, P9, P10, P11)	
DFH-5	*C. parvum* IId19GA1 (H39)	*C. andersoni* (B8)	E (B7)
DFH-8		*C. scrofarum* (P14)	AI + E (P13)
DFH-9		*C. scrofarum* (P27)	E (P27, P28)
SF-10			AI (P46); E (P54)
GA-4			E + B (Sh20); E (Sh15, Sh16, Sh17, Sh18, Sh23, Sh24, Sh26, Sh27, Sh28, Sh30, Sh31, Sh37, Sh38, Sh40)
GA-8			E (Du10)
YHA-2			E (E58, E59)
YHA-3		*C. canis* XXa4 (F8, F12)	D (F36)
YHA-7			E (Sh56, Sh58)
CF-1		*C. scrofarum* (P95, P105, P117, P118); *C. suis* (P97, P99, P110)	

*H* human, *B* cattle, *C* chicken, *Du* duck, *E* goose, *F* fox, *P* pig, *S* goat, *Sh* sheep, *CF* Changfa village, *DFH* Dongfanghong village, *GA* Gongan village, *SF* Shuangfa, *YH* Yuhe village, *YHA* Youhao village

No *G. duodenalis* infection in humans

## Discussion

The prevalence of *Cryptosporidium* or *G. duodenalis* varies in humans and animals between countries and even between regions of the same country. In the present study, the prevalence of *Cryptosporidium* was 0.9% in humans who lived in rural areas, which was lower than the rates reported in most previous studies. For example, in the Yi Autonomous Prefecture in southwestern China, a region with a mild climate belonging to the subtropical humid zone, the prevalence of *Cryptosporidium* (12.0%, 74/615) was high among the village residents [26]. Meanwhile, a higher prevalence of *Cryptosporidium* was also found in the general population in other countries, such as 69.6% in Mexico [27]. Furthermore, humans were not found to be infected with *G. duodenalis* in the present study, indicating a lower prevalence compared with previous studies, such as 6.1% in the Hui ethnic group in Qinghai Province [28] and 8.2% in rural areas of Sichuan Province [29]. Comparatively, a higher prevalence of *G. duodenalis* was reported in other countries, particularly in Africa, with a prevalence of 11.7% among the agricultural population in Morocco [30], 29.0% in rural districts in northern Ethiopia [31], and as high as 34.6% in people in Egypt [32]. The prevalence of the two parasites may be influenced by weather and climate conditions; low temperatures and dry climate present may reduce the chance of *Cryptosporidium* and *G. duodenalis* transmission [33, 34]. In this study, only one human-derived *Cryptosporidium*-positive isolate was obtained, and it was collected during the warm and rainy month of August. Therefore, the low prevalence could be attributed to the majority of samples being collected during the fall and winter seasons in northeast China.

The prevalence of *Cryptosporidium* and *G. duodenalis* exhibits significant variation in animals between and within countries worldwide. For instance, in domestic mammals, the prevalence of *Cryptosporidium* ranged widely, from 0.1% to 100% in pigs and from 0.0% to 100% in cattle [15]. In poultry, such as chickens, prevalence also displayed considerable variation. Prevalence had been reported to be 0.5% in Iran [35], while in Algeria, the prevalence reached as high as 34.4% [36]. In the present study, *Cryptosporidium* was detected in various domestic animals (pigs, cattle, foxes, and chickens) with prevalence ranging from 0.4% in chickens to 8.8% in pigs. Notably, *Cryptosporidium* was identified in chickens for the first time in Heilongjiang Province, with lower prevalence (0.4%) compared with other Chinese provinces. For example, prevalence in chickens from Guangdong Province was 13.2% [37], followed by Hubei Province with 10.2% [38]. For *G. duodenalis*, it was also detected in a variety of domestic animals (pigs, cattle, sheep, foxes, ducks, and geese), with prevalence ranging from 2.2% in foxes to 28.8% in sheep. To our knowledge, *G. duodenalis* was identified in foxes for the first time in China.

In this study, six *Cryptosporidium* species were identified, including *C. parvum*, *C. canis*, *C. meleagridis*, *C. suis*, *C. scrofarum*, and *C. andersoni*. In a phylogenetic analysis, the sequence of *C. parvum* obtained from a human sample in this study clustered with those from cattle (MF671870, MK426796) in one branch. Simultaneously, the sequences of *C. canis*, *C. meleagridis*, *C. suis*, *C. scrofarum*, and *C. andersoni* obtained from animal samples clustered with human-derived sequences KT74981, AY166839, MH187877, EU331243, and KF826313, respectively, on the corresponding branches. The findings suggest these *Cryptosporidium* species have a zoonotic potential, and animals may serve as a reservoir for humans infected with *Cryptosporidium* (Fig. 2) [39, 40].

Further, *C. parvum* and *C. canis* subtyping at the *gp60* gene revealed the subtype as IIdA19G1 and XXa4, respectively. The most investigated *Cryptosporidium* implicated in zoonotic transmission is *C. parvum*, with approximately 20 subtype families identified. Among them, the IIa and IId were defined as zoonotic parasites [41]. In China, IIdA15G1 and IIdA19G1 were found to be the dominant *C. parvum* IId subtype in calves, and the IIdA19G1 has been reported in eight provinces, one autonomous region, and three municipalities [42–50], including Heilongjiang Province. Moreover, this subtype has caused outbreaks of *Cryptosporidiosis* in neonatal calves on one dairy farm in Jiangsu Province [48]. Meanwhile, human infections with IIdA19G1 were mostly reported in Europe, the Middle East, and New Zealand [51–53]. In China, it was also identified in four hospitalized children and two patients with human immunodeficiency virus (HIV) [54, 55]. These findings suggest that neonatal calves may act as a reservoir for the transmission of human *Cryptosporidium* infections [56]. However, in the present study, *C. parvum* subtype IIdA19G1 was detected in a 54-year-old non-diarrheal male participant, which is the first reported in humans in Heilongjiang Province. In contrast, this subtype was not found in the cattle of this family. On the basis of current data, it was unclear whether humans can be infected with *C. parvum* IIdA19G1 through cattle. Therefore, more human and domestic animal samples are needed to identify the potential animal reservoirs of human infection.

*C. canis* is the most frequently identified species in dogs worldwide, and it is also found in foxes, coyotes, minks, mongoose, raccoon dogs, cattle, and sheep [57, 58]. Notably, human cases of cryptosporidiosis caused by *C. canis* have been reported in immunocompetent humans in the USA, Ethiopia, and Peru and immunocompromised individuals in Jamaica and Peru [59, 60]. Due to the zoonotic nature of *C. canis*, people should be aware of the potential zoonotic transmission of cryptosporidiosis. By using the newly developed subtyping tool, *C. canis* has been classified into seven known subtype families (IIIa–IIIg) [18, 61]. In the present study, two *C. canis* isolates derived from foxes were identified as XXa4, which was the first reported worldwide. Wang et al. reported that XXa was only detected in raccoon dogs, while XXg was only in foxes, indicating the existence of host adaptation in *C. canis* among fur animal species [61]. Our results are inconsistent with those of previous studies, and due to the limited number of positive samples, we are unable to provide a satisfactory explanation for the host adaptation. Therefore, a substantial number of samples from foxes are needed to validate this subtype of the host adaptation. Despite no *C. canis* infection being detected in owners in the same households in our study, the zoonotic potential of *C. canis* should not be overlooked.

*C. meleagridis* has been detected in various avian hosts, such as pigeons, quails, chickens, turkeys, and so on [62–65]. However, it has also been identified in humans in several low- and middle-income countries, including in Africa and Asia [55, 66, 67]. To date, *C*. *meleagridis* has ten known subtype families (IIIa–IIIi), with IIIb among the most common. Molecular studies have revealed that identical *C. meleagridis* subtypes were identified in humans and chickens in the same location in Sweden, suggesting cross-species transmission of *C. meleagridis* between birds and humans [38, 39]. In this study, *C. meleagridis* the species was found in a single chicken, and this isolate was not successfully subtyped. Noteworthy, chickens may act as a source of infection or a mechanical vector by shedding *C. meleagridis* oocysts into the environment [37].

In the present study, *C. suis* and/or *C. scrofarum* were only identified in pigs, which are the type hosts for both these species [40, 68, 69]. Moreover, studies demonstrated that *C. suis* and *C. scrofarum* could also infect humans. *C. suis* was identified in individuals with diarrhea in England, [70], as well as in children in Cambodia [71], and in patients infected with HIV in China, Peru, and Thailand [55, 72, 73]. Therefore, pigs in study areas could pose a potential threat to human security due to asymptomatic infection and close contact with humans. In addition, *C. andersoni* was found in one cow in the present study*. C. andersoni* mostly responsible for bovine cryptosporidiosis [64], and has also been reported to infect different animals, such as mice [74], cattle, sheep, goats [75], birds [76], cats, and dogs [10]. Human infections with *C. andersoni* have been detected in several countries, including the UK [70], Malawi [71], Iran [77], and China [72], while in China, a high prevalence of *C. andersoni* in immunocompetent children and adults has been reported [72, 78]. In the present study, *C. andersoni* was identified in one cow, which remains a severe threat to susceptible animals (cattle, sheep, and goats) and humans.

For *G. duodenalis*, assemblages A, D, and E were identified in domestic animals in our study. Additionally, two mixed infection cases were found, with A + E in one pig and B + E in one sheep. Among them, assemblage E was the most prevalent (Tab S2). Several studies have suggested that assemblage E is strongly associated with host specificity, particularly in cloven-hoofed livestock [79–82]. However, more than 50 human cases of assemblage E have been identified in recent years in Brazil, Egypt, Vietnam, Australia, and New Zealand [53, 81–84], highlighting its potential for zoonotic transmission. In this study, assemblage E was found in sheep (16/27), pigs (3/27), cattle (5/27), ducks (1/27), and geese (2/27). Despite cohabitation between humans and these domestic animals, assemblage E was not detected in humans. Assemblage E is frequently encountered in hoofed mammals and it has previously been reported in fecal droppings from brown- and black-headed gulls, geese, and cormorants [85]. In the meantime, it is reported that *Giardia* cysts have been identified in the feces of migratory Canada geese. These geese are known to follow cattle and consume undigested plant material found in cattle feces [86]. Hence, it is plausible that the presence of assemblage E in ducks and geese may be attributed to the contamination of their environment from cattle or sheep feces, as these animals were also found to be infected with assemblage E in the current study. Additionally, previous research also showed that the occurrence of *Giardia* cysts in bird feces might indicate mechanical transmission rather than established infections [12, 87].

Assemblages D is predominantly associated with dogs, foxes, coyotes, and seals [10, 88] and has also been identified in pigs and cattle [89, 90]. In rare instances, it has been reported in cats [91]. In the present study, assemblage D was only detected in a fox. Notably, assemblage D has also been reported in humans, and to date, no more than 16 cases of human giardiasis have been found in Europe, Egypt, Germany, and Thailand [11, 32, 92, 93]. These results revealed that the host-adapted assemblage D was no longer confined to specific hosts, and possessed a broader host range than previously believed.

Assemblages A and B are responsible for most giardiasis cases in humans. Assemblage B was more commonly reported in Asia, Oceania, Europe, and Africa. However, in South America and the Middle East, assemblage A is more common [1, 94–96]. Among farm animals, such as cattle, sheep, and goats, assemblage A has been found to have some occurrence and assemblage B occasional occurrence [1]. In the assemblages A: AI is considered zoonotic, AII is mainly found in humans, and AIII is exclusively found in animals [22]. In the present study, assemblage AI was confirmed in one pig. By sequence analysis, assemblage AI sequence compared with reference sequence download from GenBank had a single nucleotide polymorphism (SNP). However, another pig exhibited a mixed infection of assemblage AI and E, with assemblage E having a SNP. Furthermore, one sheep showed a mixed infection of assemblage E and B. However, assemblages B, D, and E were compared with corresponding reference sequences from GenBank, which revealed no SNPs.

In the present study, an overlap of subtype between animals and their owners in *Cryptosporidium* spp. and *G. duodenalis* was not found, which suggested a limited role of domestic animals in the human cryptosporidiosis or giardiasis in the studied population. Nevertheless, it is necessary to pay special attention to the zoonotic potential of these species, especially in children and immunocompromised individuals who have close contact with domestic animals.

## Conclusions

The present study describes the prevalence, species, and subtypes of *Cryptosporidium* and assemblage and sub-assemblage of *G. duodenalis* in humans and domestic animals in nine villages in Heilongjiang Province. *C. parvum* was discovered in a human, and *C. canis*, *C. meleagridis*, *C. suis*, *C. scrofarum*, and *C. andersoni* were identified in domestic animals. These species are zoonotic *Cryptosporidium* species. However, the *G. duodenalis* assemblages A, B, D, and E were only found in domestic animals. Assemblages A and B are considered potentially zoonotic, while the other assemblages have also been reported in humans. The presence of these *Cryptosporidium* species and *G. duodenalis* assemblages in animals poses a potential risk to human health, especially in areas where humans have close contact with infected animals. Therefore, public education programs should be implemented to raise awareness among villagers regarding the potential transmission of parasites from domestic animals and the importance of hygiene in disease prevention.

Furthermore, it is recommended that veterinarians and physicians should implement effective control measures to reduce the burden of these two parasitic diseases. Their involvement will contribute to enhancing the overall health and well-being of the rural community in their coexistence with domestic animals.

### Supplementary Information

**Additional file 1: Table S1.** Homology analysis of the *SSU* rRNA genes of *Cryptosporidium*-positive samples at the nucleotide level.

**Additional file 2: Table S2.** Assemblage distributions of *G. duodenalis* in domestic animals in Heilongjiang Province.

**Additional file 3: Table S3.** Homology analysis of the *SSU* rRNA, *bg*, *gdh*, and *tpi* genes of *G. duodenalis*-positive samples at the nucleotide and amino acid levels.

## Data Availability

All data generated or analyzed during this study are included in this published article. The nucleotide sequences have been deposited in GenBank database under the accession numbers: OR357660 to OR357666 and OR353406 to OR353407 (*Cryptosporidium*); OR359371 to OR359372, OR353408 to OR353416, and OR360610 to OR360618 (*G. duodenalis*).

## Abbreviations

CF: Changfa village
DFH: Dongfanghong village
GA: Gongan village
QJ: Qianjin village
SF: Shuangfa village
XL: Xinglong village
XF: Xianfeng village
YH: Yuhe village
YHA: Youhao village
*SSU* rRNA: Small subunit ribosomal ribonucleic acid
NJ: Neighbor-joining
PCR: Polymerase chain reaction
bp: Base pair
SNPs: Single nucleotide polymorphisms
*bg*: ß-giardin
*gdh*: Glutamate dehydrogenase
*tpi*: Triosephosphate isomerase
*gp60*: 60-KDa glycoprotein
