# Supplementary material for: Molecular characterization and zoonotic potential of Cryptosporidium spp. and Giardia duodenalis in humans and domestic animals in Heilongjiang Province, China
Source: Parasit Vectors. 2024 Mar 25;17:155. doi: 10.1186/s13071-024-06219-3 (PMC10964600; doi:10.1186/s13071-024-06219-3)
Supplement: Supplementary file 1 — Additional file 1: Table S1. Homology analysis of the SSU rRNA genes of Cryptosporidium-positive samples at the nucleotide level. [file 13071_2024_6219_MOESM1_ESM.docx]

**Additional file 1: Table S1.** Homology analysis of the *SSU* rRNA genes of *Cryptosporidium*-positive samples at the nucleotide level

| Specie | Host (n) | Accession no.^a^ /product size (bp) | Accession no.^b^ (Host) | Homology | Nucleotide^c^ |
| --- | --- | --- | --- | --- | --- |
| *C. parvum* | Human (1) | OR357663/832 | MN914084 (Human); MK426796 (Pre-weaned calves); MK491508 (One-humped camel); MK241967 (Cattle); MF671870 (Dairy cattle); KU679364 (Hedgehog); KT151548 (Quail); KT151531 (Wild duck); OP102684 (Mice); AB513875 (Calf) | 100% |  |
| *C. scrofarum* | Pig (1) | OR357664/807 | MH174663 (Tibetan pig); ON149811 (Pig); KU668893 (Wild boar) | 99.9% | C(C→T)T |
|  | Pig (6) | OR357665/795 | EU331243 (Human); KU668893 (Wild boar); KU668895 (Xiang pig); MH178036 (Landrace pig); MT071828 (Pig) | 100% |  |
| *C. suis* | Pig (7) | OR357666/816 | MH187877 (Human); MH178034 (Landrace pig); JF710259 (Pig); OP090505 (Cattle); OQ520115 (Flies); AB449862 (River water) | 100% |  |
| *C. meleagridis* | Chicken (1) | OR357662/802 | MT757970 (Human); MN410718 (Parrot); MF498749 (Raw water); KT151551 (Red jungle fowl); MW090930 (Waste water); EU814432 (Pigeon); EF158460 (Broiler chicken); AF112574 (Turkey); KY352486 (Greylag goose); MW783462 (Budgie); EU827311 (Chicken); JX416368 (Dairy cattle); MH062745 (Quail) | 100% |  |
| *C. andersoni* | Cattle (1) | OR357660/792 | KF271454 (Human); MN379942 (Whooper swan); OP861843 (Dairy cattle); FJ608606 (Sheep); HM002493 (Water buffalos) | 100% |  |
| *C. canis* | Fox (2) | OR357661/830 | KT749817 (Child); MN696800 (Dogs); MN238765 (Raccoon dogs); MN238764 (Blue foxes); MN235856 (Mink) | 100% |  |

^a^ Accession no. indicating the nucleotide sequences obtained in this study

^b^ Accession no. of the reference sequences, which had the highest similarity with the representative sequences obtained in the present study

^C^ The nucleotide change (in brackets) represents the change from the reference sequence to the representative sequence obtained in the present study
