# Supplementary material for: Molecular characterization and zoonotic potential of Cryptosporidium spp. and Giardia duodenalis in humans and domestic animals in Heilongjiang Province, China
Source: Parasit Vectors. 2024 Mar 25;17:155. doi: 10.1186/s13071-024-06219-3 (PMC10964600; doi:10.1186/s13071-024-06219-3)
Supplement: Supplementary file 2 — Additional file 2: Table S2. Assemblage distributions of G. duodenalis in domestic animals in Heilongjiang Province. [file 13071_2024_6219_MOESM2_ESM.docx]

**Additional file 2: Table S2**. Assemblage distributions of *G. duodenalis* in domestic animals in Heilongjiang Province

| Isolate code | *SSU* rRNA | *bg* | *gdh* | *tpi*^a^ |
| --- | --- | --- | --- | --- |
| E58 |  | E |  |  |
| E59 |  | E |  |  |
| F36 |  | D | D |  |
| B2 |  | E | E |  |
| B3 | E | E | E | E |
| B7 | E | E | E |  |
| B18 | E | E |  | E |
| B19 | E | E | E |  |
| P13 |  | E | A | E |
| P27 |  |  | E |  |
| P28 | E |  |  |  |
| P46 |  |  | A |  |
| P54 |  |  | E |  |
| Y10 |  |  | E |  |
| Sh15 | E |  |  |  |
| Sh16 | E |  |  |  |
| Sh17 | E |  |  |  |
| Sh18 | E |  |  |  |
| Sh20 | E |  |  | B |
| Sh23 | E |  |  |  |
| Sh24 | E |  |  |  |
| Sh26 | E |  |  |  |
| Sh27 | E |  |  |  |
| Sh28 | E |  |  |  |
| Sh30 | E |  |  |  |
| Sh31 | E |  |  |  |
| Sh37 | E |  | E |  |
| Sh38 | E |  | E | E |
| Sh40 | E |  |  | E |
| Sh56 | E |  |  |  |
| Sh58 | E |  |  |  |

^a^ At the *tpi* locus, all *G. duodenalis*-positive samples were only sequenced successfully using assemblage-specific primers
