# Supplementary material for: Molecular characterization and zoonotic potential of Cryptosporidium spp. and Giardia duodenalis in humans and domestic animals in Heilongjiang Province, China
Source: Parasit Vectors. 2024 Mar 25;17:155. doi: 10.1186/s13071-024-06219-3 (PMC10964600; doi:10.1186/s13071-024-06219-3)
Supplement: Supplementary file 3 — Additional file 3: Table S3. Homology analysis of the SSU rRNA, bg, gdh, and tpi genes of G. duodenalis-positive samples at the nucleotide and amino acid levels. [file 13071_2024_6219_MOESM3_ESM.docx]

**Additional file 3: Table S3.** Homology analysis of the *SSU* rRNA, *bg*, *gdh*, and *tpi* genes of *G. duodenalis*-positive samples at the nucleotide and amino acid levels

| Target gene | Assemblage/Sub-assemblage (Host/no.) | Accession no.^a^ /product size (bp) | Accession no.^b^ (Host) | Homology | Codon/amino acid |
| --- | --- | --- | --- | --- | --- |
| *SSU* rRNA | E (Pig/1, Cattle/4, Sheep/15) | OR359371/294 | DQ157271 (Dairy calf); AY826208 (Sheep); OQ818654 (Pig) | 100% |  |
|  | E (Sheep/2) | OR359372/294 | DQ157271 (Dairy calf); AF199448 (Goat); AY826208 (Sheep); OQ818654 (Pig); OQ818652 (Horse); KR048491 (Takin) | 100% |  |
| *bg* | D^#1^ (Fox/1) | OR353408/511 | MT713335 (Dog) | 100% |  |
|  | E^#1^ (Goose/1) | OR353412/514 | OM653985 (Cattle) | 100% |  |
|  | E^#2^ (Cattle/2) | OR353411/511 | OM653968 (Cattle); OM810206(Racoon dog); MK610387 (Sheep); KT922248 (Calf); EU189361 (Goat) | 100% |  |
|  | E^#3^ (Cattle/2, Pig/1) | OR353409/511 | KT369770 (Cattle); KY633470 (Yaks) | 100% |  |
|  | E^#4^ (Cattle/1, Geese/1) | OR353410/499 | MN833266 (Sheep); OM653969 (Cattle) | 100% |  |
| *gdh* | A/AI^#1^ (Pig/1) | OR360610/508 | KC960643 (Sheep); MT319049 (Two-humped camel); MN174853 (Race horse); LC437366 (Domestic dog); MH051905 (Reindeer) | 100% |  |
|  | A/AI^#2^ (Pig/1) | OR360611/499 | MK645798 (Tan sheep); MF671911 (Chipmunk); MN174853 (Race horse); MF169199 (Horse); EF507600 (Cat) | 99.8% | G(A→T)G/V→E |
|  | D^#2^ (Fox/1) | OR360612/508 | KR855633 (Dog) | 100% |  |
|  | E^#5^ (Cattle/1, Duck/1) | OR360613/466 | KY432839 (Cattle) | 100% |  |
|  | E^#6^ (Cattle/1, Pig/1) | OR360614/508 | KT698969 (Dairy cattle); MH794178 (Sheep); MN833268 (Goat); MK982483(Children) | 100% |  |
|  | E^#7^ (Cattle/1) | OR360615/502 | MN833273 (Sheep) | 100% |  |
|  | E^#8^ (Cattle/1)  E^#9^ (Sheep/2) | OR360616/508 | MK442909 (Sheep) | 100% |  |
|  |  | OR360617/505 | MK642911 (Dairy cattle); MK890218 (Cattle) | 100% |  |
|  | E^#10^ (Pig/1) | OR360618/508 | AY178741 (Pig); MG820464 (Calf) | 100% |  |
| *tpi*^c^ | B^#1^ (Sheep/1) | OR353413/376 | JX994246 (Human); KT372238 (Rabbit); KM067095 (Dairy cattle); JQ928712 (Sheep); HQ603781 (Waste water) | 100% |  |
|  | E^#11^ (Cattle/1) | OR353414/366 | MH893667 (Dairy cattle); N773559 (Holstein cattle) | 100% |  |
|  | E^#12^ (Cattle/1, Sheep/2) | OR353415/366 | KU378634 (Human); MH230886 (Yak); MK890222 (Cattle); MK252662 (Dairy cattle); MK442912 (Sheep); MG602957 (Goat) | 100% |  |
|  | E^#13^ (Pig/1) | OR353416/373 | MK442915 (Sheep); MH158499 (Cattle); MG976832 (Goat); OQ934102 (Pig); MT319058 (Two-humped camel) | 99.7% | A(G→A)G |

^a^ Accession no. indicating the nucleotide sequences obtained in this study

^b^ Accession no. of the reference sequences, which had the highest similarity with the representative sequences obtained in the present study

^#^ indicating the representative sequences obtained in the present study

^c^ At the *tpi* locus, all *G. duodenalis*-positive samples were only sequenced successfully using assemblage-specific primers
